# Predicting ‘Brainage’ in the Developmental Period using Structural MRI, Morphometric Similarity, and Machine Learning

**DOI:** 10.21203/rs.3.rs-2583936/v1

**Published:** 2023-02-28

**Authors:** Daniel J. Griffiths-King, Amanda G. Wood, Jan Novak

**Affiliations:** Aston University; Murdoch Children’s Research Institute; Aston University

**Keywords:** MRI, Brain Development, Healthy, Structural MRI, Morphometric Similarity, Brain-Age

## Abstract

Brain development is regularly studied using structural MRI. Recently, studies have used a combination of statistical learning and large-scale imaging databases of healthy-children to predict an individual’s age from structural MRI. This data-driven, ‘brainage’ typically differs from the subjects chronological age, with this difference a potential measure of individual difference. Few studies have leveraged higher-order or connectomic representations of structural MRI data for this brainage approach. We leveraged morphometric similarity as a network-level approach to structural MRI to generate predictive models of age. We benchmarked these novel brain-age approaches using morphometric similarity against more typical, single feature (i.e. cortical thickness) approaches. We showed that these novel methods did not outperform cortical thickness or cortical volume measures. All models were significantly biased by age, but robust to motion confounds. The main results show that, whilst morphometric similarity mapping may be a novel way to leverage additional information from a T1-weighted structural MRI beyond individual features, in the context of a brain-age framework, morphometric similarity does not explain more variance than individual structural features. Morphometric similarity as a network-level approach to structural MRI may be poorly positioned to study individual differences in brain development in healthy individuals.

## Introduction

Developmental neuroscience has embraced neuroimaging studies of brain structure to characterize brain maturation and to understand how this gives rise to cognitive development. Developmental neuroimaging studies have highlighted distinct developmental trajectories of specific cortical tissues such as white matter (WM) and grey matter (GM), across different regions of the cortex [1]. The volume of cortical GM specifically shows an ‘inverted U’, nonlinear trajectory [1–4], with pre-pubertal expansion of the cortical GM [5] followed by a post-pubertal sustained loss of GM volume (despite synaptic density plateauing after puberty according to molecular and cellular evidence [5]). Brain maturation has specific regional trajectories; peak GM density and reductions in GM volume occur earliest in primary function areas, somatosensory and primary motor cortices, and latest in higher-order association areas, dorsolateral prefrontal cortex and superior temporal gyrus for instance [1]. Cortical thickness maturation also shows a similar trajectory, with generalised reductions over time [6–8], in line with what would be expected from models of synaptic pruning and myelination [8]. These longstanding findings show, given these measures vary as a function of age, that an individual’s chronological age may be deduced from an MRI scan of their brain.

This is the premise of the brainage framework, the idea that multivariate patterns of brain structure in large samples of MRI from healthy children are related to age and, by using data-driven or machine learning approaches, that association can be learnt. By applying these learnt patterns to new data, we can predict the age of an individual based on their MRI (see [9, 10] for review). This apparent age, or more commonly brain age, is akin to a reading age for instance; it reflects the current observed status of the brain in terms of morphometry in comparison to ‘typical’ norms of brain structural development. The brainage of any individual is unlikely to be perfectly aligned to their chronological (actual) age. Differences between brainage and chronological age may reflect normal-variation or individual differences between children or, in more extreme differences, represent perturbation of brain development, for instance if we applied these models to diseased cohorts (i.e. [11]). This metric of perturbation is typically referred to in the field as the brain-age Δ, the calculated difference between chronological and apparent/predicted brain age. [9,11].

In the case of diseased populations, this measure allows us to estimate the perturbation that the disease state has upon brain development. Brain development (specifically grey matter change) follows highly ‘programmed’trajectories [5, 12–14], (driven in part by genetics [15–18]). Therefore, neurological disruption to the ongoing development of the brain during this period is likely to potentially symptomatic, impacting on future brain and cognitive maturation. Therefore, an approach for quantifying typical brain maturation will likely hold benefit in understanding atypical developmental patterns that hold clinical implication [19, 20].

In recent years, data-driven estimations of the brain’s apparent age have been calculated using machine-learning approaches to detect latent patterns associated with age across several neuroimaging modalities (including structural (sMRI), diffusion (dMRI) and functional MRI (fMRI)). Utilising machine learning approaches in this way, can consider the multivariate complexities of the neurodevelopmental trajectories of these meso-scale measures. Previous approaches utilising structural MRI (i.e. T1w sMRI) in paediatrics [19, 21, 22] have achieved comparable prediction accuracies to those multimodal studies incorporating additional modalities with sMRI [23, 24] (although, methodological differences preclude meaningful direct comparison across these paediatric studies). Given sMRI is more readily acquired across clinical and research contexts, and comparable performance in brainAge estimation, sMRI is a suitable approach for this research.

However, using regional-level data as independent features to predict age may ignore potentially relevant, higher-order multicolinearities between regions. Connectomic approaches [25], that consider the brains’s network-level organisation, may therefore hold greater potential in predicting *brain-age*. However, diffusion and functional connectomes have limitations, such as noise and variability in processing pipelines, which may introduce error in the normative models [26]. Also, the MRI sequences required to generate fMRI and dMRI datasets have long acquisition times and may therefore be less tolerable in clinical populations and paediatrics [27]. Thus, this study utilise connectomic approaches to sMRI to predict brainage in pediatric cohorts.

However, relatively few studies leverage connectomics-level analyses of sMRI data for brain-age prediction. Corps and Rekik [26] utilised morphometric data (curvature, cortical thickness, sulcal depth) to produce multi-view morphological brain networks. Briefly, they investigated multiple feature-networks where ‘connections’ are the dissimilarity (absolute difference) between regions in terms of absolute feature values. This produced multiple feature networks as predictive variables. No previous studies have leveraged network-level approaches to sMRI to generate single morphometric networks as variables in predictive models of age.

In the current paper, we employed a morphometric similarity mapping approach [28] to combine multiple features into a single network, capturing higher-order morphometric organisation across the cortex. Previously these networks have been shown to be sensitive to neurodevelopmental abnormalities [29]. Specifically, this paper generated normative brain-age models using connectomic approaches to sMRI, as outlined in King and Wood [27], leveraging both network-level approaches whilst restricting necessary MRI sequences to a T1w sMRI. This approach may better account for absolute dissimilarity due to scaling (as in Corps and Rekik [26]) and instead capture those relationships that are indicative of coordinated cortical development and maturation [28].

The current study evaluates the use of T1w morphometric similarity mapping in predicting brain age in a cohort of typically developing children. This study answers whether brain age prediction methods are more accurate when using morphometric similarity network-level data. To investigate this, we benchmark novel brain-age approaches using morphometric similarity against more typical single morphometric feature approaches across several computational experiments.

## Results

### Dataset

To evaluate these novel methodologies, we employed data from healthy controls from the open-access Autism Brain Imaging Data Exchange cohort (ABIDE, Di Martino, Yan [30]) data from the Preprocessed Connectome Project (PCP Bellec, Yan [31], for full details see Pre-processed Connectome Project website http://preprocessed-connectomes-project.org/). Healthy controls were included that were > 17 years old and met strict quality control criteria (outlined below). The subsequent dataset consisted of 327 healthy controls, with a mean age of 12.4 ± 2.5 yrs (see Table 1). We utilized the Freesurfer [32] processed outputs supplied by the PCP. This provides cortical morphometry measures across regions of the Desikan-Killany atlas [33].

### Model Evaluation

**Figure 1:**
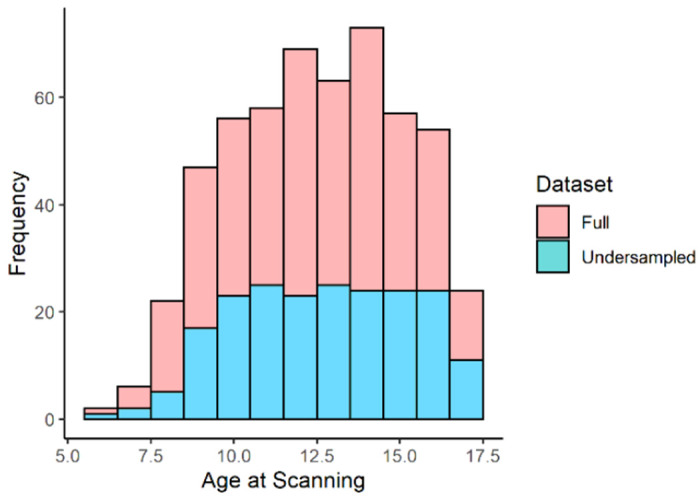
Age of participants in the entire cohort versus those in the training cohort. This graph highlights the undersampling of the training set based on age. This was less successful at the extreme ‘tails’ of childhood (approximately less than 8yrs and greater than 16.5yrs) where less data was available to sample from.

To evaluate predictive capacity of the morphometric similarity network (MSN), the ABIDE cohort were divided into training, validation and test sets in the ratio of 5:1:2 (n = 204, n = 41 & n = 82 respectively). Selection of the training-set was pseudo-random to enable under sampling based on age (see Fig. 1).

Prediction utilized 10 different feature-sets (See Table 2); i – vii) each of the individual morphometric features, viii) all features, ix) nodal-level strength of the MSN and x) edge-level weights of the MSN, with each model having chronological age at scanning as the dependent variable. Across models, performance was evaluated based upon reducing mean absolute error (MAE) and maximizing predictive R^2^.

### Training and Validation Results

Brain-age prediction was conducted across two, kernel-based regression approaches; a) Gaussian Processes Regression (GPR) and b) Relevance Vector Regression (RVR).These were selected as both are commonly used in the literature [9, 10, 34], and non-linear and/or kernel-based algorithms typically outperform linear approaches (likely due to the multicollinearity in morphometric measures [35, 36]. Two different kernels were tested for each algorithm: a) laplacedot (Laplace radial basis kernel) and b) RBFDot (Gaussian radial basis function). Algorithm and kernel selection was conducted based upon performance when trained on the training data and evaluated on the validation set.

Table 3 highlights the performance of each model in both the training and validation sets. For all feature sets, gaussian processes regression (paired with either the laplacedot or RBFdot) seemed to perform best on the validation set. The model (algorithm + kernel) which performed best on the validation set for each feature set was evaluated on the independent test set to estimate performance for each feature set.

### Model Evaluation on Test Set

**Figure 2:**
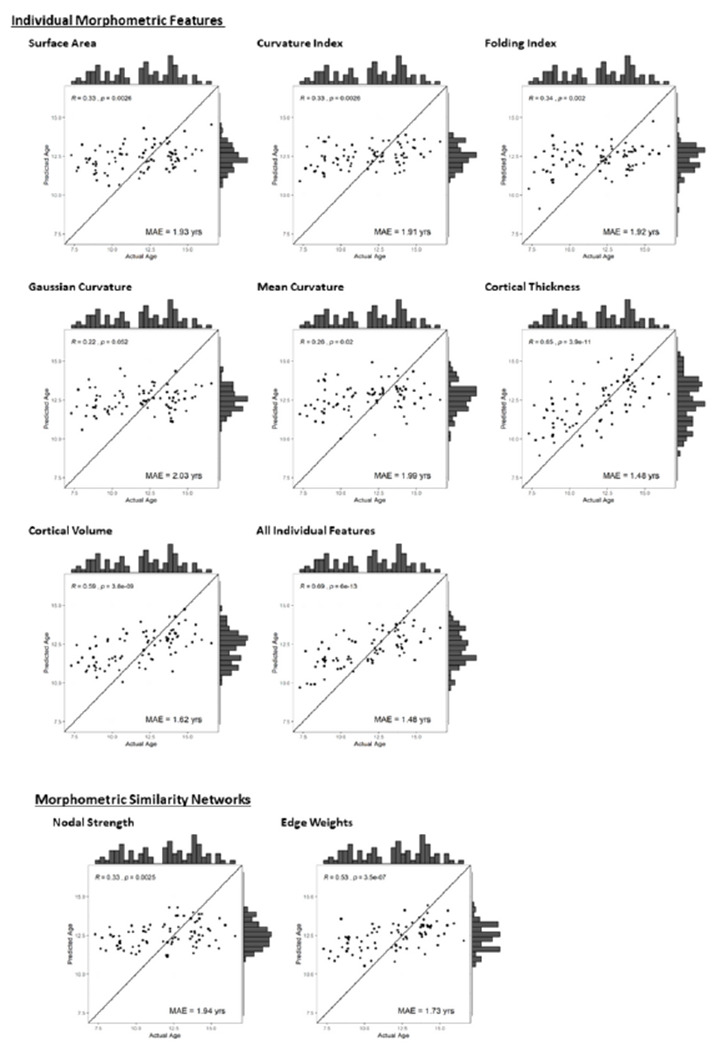
Performance of brain age prediction on testing cohort, for each of the feature sets, including a) individual morphometric features and b) network features based on the MSN. Chronological age is plotted against the age predicted by the model. Plotted line is where actual age = predicted age (x = y), which would represent perfect prediction.

When models were evaluated on the independent test set, training and validation sets were combined to provide a larger training set. Models trained on this larger training set (n = 245) were then tested on the test set. Table 4 highlights the results of this model testing, with data plotted in Fig. 2. Evaluations suggest that Gaussian and mean curvature performed poorest, with prediction worse than a model of just the mean (R^2^=−0.05 &−0.09 respectively). MSN edge weights, cortical volume, thickness and all individual features performed strongest (R^2^ = 0.19, 0.29, 0.37 & 0.39 respectively). Based on random resampling of the data (training/validation/testing), we calculated mean predicted R^2^ of models and 95% confidence intervals (CI) of these values. Only models based upon MSN edge weights, cortical volume, thickness and all individual features had 95% CI that did not cross predictive R^2^ = 0. Performance across the resampling for these models was still variable, with the range of the 95% CI around .21.

We also produced null models by permuting age in the training and validation groups and evaluating on actual testing data. The mean predictive R^2^ values of the resampled models, and the distribution of R^2^ values from the permuted ‘null’ cases allowed calculation of *p*-values, where the models performed above random noise in the data. Again, only models based upon MSN edge weights, cortical volume, thickness and all individual features produced models which performed significantly above null models.

### Prediction using Density Thresholded MSN

Given that correlation-derived networks may represent both ‘real’ statistical associations and potential noisy/spurious associations [37] we also tested prediction based upon the edge-level MSN, (thresholded at an individual-level) at multiple densities; from top 5% edges to 50% in steps of 5%. For all densities, in terms of both predicted R^2^ and MAE, GuassPrc outperformed the RVM algorithm on the validation set. Irrespective of kernal, prediction performed equally well (to 2dp) for all densities tested (MAE = 1.73yrs, Pred. R^2^ = .32). Given the predictive accuracy remains even when the network is made sparser, this suggests that the top 5% of edges in terms of weight are those that are most sensitive to individual differences due to age. As the performance did not change compared to the original, unthresholded network, we used the unthresholded network for the remaining analysis.

### Potential Biases in BrainageΔ

**Figure 3:**
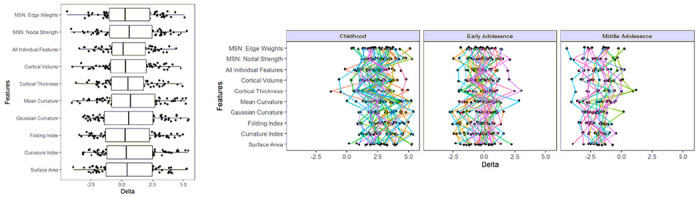
Plots showing BrainageΔ for the testing cohort; across each model based on different feature sets (left) and when divided into developmental periods of childhood (5-11yrs), early adolescence (11-14yrs) and middle adolescence (14-17yrs).

The BrainageΔ were calculated us the absolute difference between actual and predicted ages (specifically in the testing cohort). This indexes the degree to which an individual diverges from age-expected, brain development (combined with model error). As expected, due to the ‘healthy’ nature of the participants, many of these values were close to zero (across all feature sets; mean(SD) = .62(2.11), median = .44), although there was large variability. Whilst the variation of BrainageΔ was similar across models, Fig. 3 also shows that, at an individual level there was great variability in BrainageΔ between models.

#### A) Potential Biases in BrainageΔ: Age

Visual inspection of performance on test data highlighted that across many of the feature sets there was a flatter gradient in the data (actual vs predicted age) than the x = y line suggesting an overestimation of age in the younger ages and an under estimation of ages in the older age participants. This was further seen in Fig. 3 when individual BrainageΔ profiles (across models) when divided by age group (childhood, early adolescence, middle adolescence).

Age-related bias in the BrainageΔ were statistically assessed using correlation analyses. There was strong statistical evidence of (actual) age bias, with correlation between BrainageΔ and actual age across the models close to minus one in all cases (r = −.92), with all correlations being statistically significant (all results can be found in Table 5). This seemed unrelated to the models general MAE performance with the cortical thickness brainage model showing the weakest (but still significant) age bias. Given the strong bias present within all models, we controlled for age in the remaining analyses of BrainageΔ using partial correlations.

#### B) *Potential Biases in* BrainageΔ: *Sex*

Potential sex differences in BrainageΔ estimation were investigated using linear models controlling for actual age. Across all models, the effect of sex did not meet significance.

#### C) Potential Biases in BrainageΔ: Motion

To evaluate potential bias in the models from motion we used the Entropy Focus Criterion (EFC [38]) as a proxy for motion derivable from T1w images. EFC uses the Shannon entropy of voxel intensities to typically quantify the amount of motion present [39], specifically through the sensitivity to motion-induced artifacts (e.g., ghosting and blurring induced by head motion). MRI autofocusing techniques based on EFC optimisation have been shown to reduce motion artifacts effectively [38].

Average correlation between BrainageΔ and EFC across the models was close to zero (r = − .03), with no correlation reaching statistical significance (all results can be found in Table 5).

### Exploratory relationship with cognition

To evaluate BrainageΔ as a putative measure of meaningful variation due to individual differences, we investigated relationships between BrainageΔ and individual variation in IQ using partial correlations (using actual age as a confound to address age-bias in the BrainageΔ measure). A limited number (n = 56) of children in the test sample had valid measures of IQ

### Combining Models for Brainage Prediction

Exploratory analyses were conducted to combine the feature sets from the best performing Brainage models to investigate whether models provided incremental increase in Brainage prediction by predicting unique variance in age. Combining cortical thickness, cortical volume and MSN edge weights, as the best performing individual features using gaussian processes regression (with rbfdot kernel), training on the full training sample (training plus validation samples) resulted in comparable performance to the best performing models seen in Table 4 (MAE = 1.06 yrs, Pred R^2^ = .74). On the testing sample, performance dropped significantly (MAE = 1.59yrs, Pred. R^2^ = .31), performing better than the Cortical volume and MSN weight models but still outperformed by the cortical thickness model. The Brainage delta estimates from this model (thickness + volume + MSN edge weights) were still biased by age (pearson’s *r*=−.93, *p* = < .00001), with no discernable relationship to individual differences in cognition (pearson’s *r*=−.17, *p* = .23).

## Discussion

To our knowledge, this is the first study to construct brain age models derived from network-level descriptions of neuroanatomical organization across the cortex. These models using morphometric similarity as a basis for predicting chronological age did not outperform non-network models, using ‘standard’ morphometric features.

Specifically, the MSN model was outperformed (in terms of lowest MAE and highest predicted R^2^) by models which included all individual structural features, followed by cortical thickness and volumetric models. However, the MSN edge weight model, alongside these better performing models, all performed significantly better than null models on testing data, suggesting that these Brainage models are capturing ‘real’ patterns of variation indicative of age.

The best performing (individual) structural feature for age prediction in this study was cortical thickness. Conversely, in a previous report of lifespan (8-96yrs) brain-age prediction, in the 8–18 year old group, across all approaches using either cortical area, thickness or volume, the greatest performance (i.e. lowest mean prediction error) was actually seen using brain volume model [40]. However, across the six prediction techniques investigated in [40], cortical thickness models outperformed cortical volume models in 3/6 methods. This similar performance is maybe unsurprising given that volume measurements are typically derived from cortical thickness (and surface area) measurements. ‘The findings of this analysis, alongside previous reports [19–21], highlights the sensitivity of cortical thickness as an index of brain maturation.

All other tested structural features (Surface Area, Curvature Index, Folding Index, Gaussian Curvature, Mean Curvature) did not significantly outperform null models, suggesting that these models may have been overfit in the training process. This is further evidenced given that the confidence intervals for performance in these models crossed zero.

We also found that combining best performing models (cortical thickness, volume and MSN edge weights) resulted in a drop in performance compared to the cortical thickness model. Whilst not a direct statistical comparison, this suggests that these models do not capture independent variance in relation to age. This seems to disagree with previous work [19] which found that joint covariation across multiple structural features predicted variance in age independently from variance in individual features.

As well as feature sets affecting brainage estimation, the machine learning or prediction workflow is also a key factor. This study found GPR to outperform the RVR approach. These methods were selected as they have been shown to outperform other linear approaches [35], including in pediatrics [36]. On the surface, our finding seems to contradict other, comparative analyses of machine learning models in predicting brainage using morphometric data who found RVR to systematically outperform GPR [41]. However, the one scenario in which GPR did outperform RVR in [41], was in the test case with the smallest number of participants, closer to that of the sample size used here. Therefore, machine learning model will be an important consideration for future use cases.

Overall, whilst these network models of sMRI such as the MSN seem to mature as a function of age in typical neurodevelopment [28], and capture meaningful variation indicative of chronological age in the Brainage framework, these networks are not most sensitive to the changes across childhood compared to other, more simplistic features, for instance cortical thickness measures.

Currently, only two other study predicted brain age from sMRI in the ABIDE cohort [26, 42]. Using a complex network approach to T1w MRI, in 7–20 year olds, [42] achieved a MAE of 1.53 years using deep learning models. The slightly larger age range means that the MAE are not entirely comparable with the current study, although the present study has outperformed this. It is important to note that the network approach to T1w MRI in this study modelled correlation gray-levels of the image rather than structural metrics.

When BrainageΔ was calculated for the test cohort, there was great variability in of an individual’s delta values for each of the feature sets; there appeared to be little consistency in these values between models. The varying individual profiles of brain age delta has two possible explanations. Firstly, brain age delta represents the combined measure of individual variance from the expected developmental trajectory plus the error in the normative age model. It therefore may be the case that the random error in each of the models is resulting in variance in BrainageΔ, across feature sets, at the individual level. This could have potential implications for the comparison of studies utilizing the Brainage measure if there is limited consistency in these measures within an individual participant. Alternatively, a potentially more interesting explanation, is that each brainage model is indexing relevant divergences/individual differences in different aspects of cortical architecture, resulting in between model variance in BrainageΔ. This could prove to be useful in neurological conditions that influence difference aspects of brain development/organization in the paediatric brain, for instance a brain age model based upon MRI measure of white matter may be more sensitive to differences from normative brain development in acute demyelinating disorders such as multiple sclerosis. In this scenario, multiple BrainageΔ’s from different features, or even imaging modalities could be used, as potential biomarkers of clinically relevant outcomes.

However, it is difficult to statistically test each of these explanations (model error vs meaningfully different divergences) because there are a limited number of models used in any one study. Future meta-analytic research could compare within-participant brain-age delta values across feature sets, whilst controlling for the MAE of the model themselves, in order to isolate ‘real’ within-individual variation in the brain age delta measure. Future studies could also use multiple (even multi-modal) brain-age models and use the feature specific BrainageΔ’s as individual predictors in regression models, to assess unique predictive variance offered by each feature.

The results of the current study are still impressive and meaningful contributions to the field. We set the bar for evaluating performance and reproducibility exceptionally high, given that;

- we tested all models on a relatively large, hold-out test set,
- we assessed robustness of performance in terms of sampling (assessing the 95% CI of performance) and against meaningful null models and,
- we investigated correlations between BrainageΔ and biases/cognition explicitly in the testing sample.

As noted by [42], ABIDE is also a particularly challenging dataset for the estimation of brainage, due to the number of different sites and acquisition protocols. For future brainage studies of development, this high bar should at least be maintained, with future improvements seen by validating on an entirely independent dataset (for example as seen in [19]).

The current study provides potential benefit to the use of the Brainage framework in clinical populations to investigate the effect of disease states on the brain. By giving estimates of error in these predictive models, brain age delta estimates can be interpreted with the appropriate amount of caution if they do not exceed ‘healthy’ variability in brain age.

An outstanding question for future research is whether there is need for models such as morphometric similarity as the popularity for deep learning/machine learning approaches become more prevalent. [43] report the results of the Predictive Analytic Competition (2019) for predicting chronological age from structural neuroimaging. They highlight the high-performing nature of neural networks for deep machine learning within the Brainage framework. Morphometric Similarity models the covariance structure of anatomical MRI features in a way which is constrained by anatomy (either using ROIs or voxels for instance) typically using a very specific, linear approach to these covariances/similarity (Pearson’s correlation coefficients). The morphometric similarity model has been shown to capture biologically meaningful information [28] however, imposing such a model as an anatomical-prior may be redundant in analyzing larger sample sizes with machine learning approaches. The machine learning/deep learning approaches that are becoming more popular in the neuroimaging literature, when fed all the individual features which are used to construct the morphometric similarity network (as we have done here), should be able to recover any covariance between structural features (even beyond linear relationships) that is captured by the morphometric similarity network approach. This may be supported by the results reported here, with greater performance seen for a model using all features compared to the morphometric similarity models.

We performed several analyses of correlates of BrainageΔ, across meaningful outcome measures and nuisance covariates/biases. We found no relationship between BrainageΔ as a measure of individual-difference and cognition in this typically-developing cohort. This suggests that, when these models are generalized to ‘novel’ cases (in this situation the testing sample), the resultant age predictions and BrainageΔ measures do not hold information pertinent to individual differences in cognition. This replicates previous similar findings. [44] reported no significant relationship between individual-level brain-age Δ (derived from voxel-based cortical thickness, volume and surface area) and cognitive abilities (as measured by the NIH Toolbox Cognition Battery). They hypothesized that this may be due to the methods they utilised which maximized the captured age-related variance in neuroanatomical measures, and that cognition-related variance (non-age related) may be captured by a different, orthogonal pattern of neuroanatomical correlates. However other studies have also found no convincing relationship between brainage and cognition in typical developing children [21, 45]. Of those that did find a relationship in developing cohorts [46, 47], these associations were small to moderate in size and thus likely require large sample sizes to reliably detect [45].

In the case of morphometric similarity (in adults), outside of the brainage framework, we found no relationship between these measures and cognitive abilities [27], failing to replicate the findings of [28]. However, a recent study of adolescence has highlighted the predictive validity of the MSN across cognition/intelligence and psychiatric symptoms [48], and so this is still very much an open area of research.

We failed to find a relationship between EFC (as a proxy measure of motion) and estimates of brainage Δ. This is most likely due to the stringent quality control procedures applied to both the training and testing cohorts, rather than a robustness to motion artefact.

One of the biggest limitations of the current study is that, in the current brainage framework, Brain Age Δ estimates are generated at the whole brain level, with a single value representing the whole brains deviance from the typical trajectory of development/aging. This comes at the cost of regional specificity which we know can be obtained by ROI/voxel cluster driven neuroimaging analyses over and above studies of whole brain. Given that we know that neurodevelopmental patterns are spatiotemporally dynamic in nature (that is they vary in location and over time [49]), and that many of the neurological disease we are interested in studying uses the Brainage framework show distinct spatial patterns in damage/cortical changes (e.g. TBI [50], AD [51]) this limitation limits the methods utility. In a recent study [52]outlined and systematically validated a local Brainage approach using a patch-based machine learning algorithm to enable estimation of voxel-wise and regional deviations from typical developmental trajectories (albeit in an adult aging population). It is yet unclear as to what contribution morphometric similarity may play in either a deep-learning framework or in the context of regional-level predictions.

## Methods

### Ethics Statement

The data used in this research was acquired through the public Autism Brain Imaging Data Exchange (ABIDE, Di Martino, Yan [30]) database. Specifically, we used the ABIDE data release as shared by the Preprocessed Connectome Project (PCP Bellec, Yan [31]. For full details see Pre-processed Connectome Project website http://preprocessed-connectomes-project.org/). The database has de-identified all the patient health information associated with the data. A favorable ethical opinion was granted by Aston University Research Ethics Committee (UREC) for the secondary analysis of the ABIDE datasets (no. 1309).

### Materials and Data Availability

The data used in this research was acquired through the public Autism Brain Imaging Data Exchange (ABIDE, Di Martino, Yan [30]) database. Specifically, we used the ABIDE data release as shared by the Preprocessed Connectome Project (PCP Bellec, Yan [31]. For full details and access see Pre-processed Connectome Project website http://preprocessed-connectomes-project.org/). Results and metadata of the current study are available on request from Dr Griffiths-King. The R code is also available from the authors upon request, however all open-source packages used in the study are listed here: *data.table, scales, psych, ggplot2, neuroCombat, ggseg, dplyr, ggpubr, ggExtra, kernlab, ppcor, PupillometryR, tidyr.*

### Participants

The ABIDE dataset consists of a large sample of 532 individuals with autism spectrum disorders and 573 typical controls, composed of MRI (functional and structural) and phenotypic information for each subject, accumulated across 17 independent neuroimaging sites. The scan procedures and parameters are described in more detail on the ABIDE website (http://fcon_1000.projects.nitrc.org/indi/abide/). We applied four inclusion criteria to this dataset, only including subjects who; a) passed a strict MRI quality control criteria of raw structural MRI (see below), b) were recorded as controls within the ABIDE database, c) at time of scan were aged < 17 years and d) had pre-processed Freesurfer data available as part of the PCP data release. This resulted in a final n = 327. Group demographics can be seen in Table 1. The ABIDE cohort had a mean IQ of approximately 110, as measured across multiple age-appropriate IQ tests (ABIDE documentation for details).

### Data Quality Check

The PCP data release includes image quality metrics (IQMs) which provide quantitative ratings of the quality of the raw T1-weighted (T1w) MR images. These are calculated using the Quality Assessment Protocol software (QAP Shehzad, Giavasis [53]). The ABIDE dataset includes data from 17 recruitment sites, and such there is potential for ‘batch effects’ on QA metrics [39]. We used the six spatial anatomical QA measures. Hence, all QA metrics were centred (mean subtracted) and scaled (divided by standard deviation) within sites, then recoded to increased values representing greater quality. This results in metrics which can be compared between sites. For each subject, QA metrics were coded as failed if they had a Zscore below − 1.5 (indicating quality which was 1.5SD below the mean). We included subjects if they had zero or one QA metric that fell below this quality metric. Of the ABIDE cases who were recorded as a) controls and b) being younger than 17 years of age at scanning (n = 361), 14 subjects were removed due to having greater than one QA metric fall below the 1.5SD cut off (20 participants also had no Freesufer data available, resulting in the final ABIDE dataset of n = 327). Further details of the automated QA measures which are included can be found here: http://preprocessed-connectomes-project.org/abide/quality_assessment.html and http://preprocessed-connectomes-project.org/quality-assessment-protocol.

### Structural MRI Processing with Freesurfer

3D tissue segmentation and estimation of morphometric features from T1w MR images was conducted using an established pipeline (Freesurfer version 5.1; details are published elsewhere Fischl, van der Kouwe [54], see Fischl [32] for review). Briefly, T1w images were stripped of non-brain tissues [55], GM/WM boundaries were tessellated and topology was automatically corrected [56, 57]. Finally, deformation of this surface was performed, to optimally define the pial (Cerebro-spinal fluid/GM) and white (GM/WM) surfaces using maximum shifts in intensity gradients to define boundaries of these tissue classes [58–60].

### Data Harmonization

Multi-site imaging data harmonization using the neuroComBat package [61, 62], an R implementation of the ComBat method [63] for removing batch-effects (i.e. site-effects) in neuroimaging data. This was applied to the participant by ROI matrix for each morphometric feature individually, to remove site effects found in the ABIDE data, whilst protecting biological variation due to age. Fortin and colleagues have shown this approach to be effective in removing site effects in multi-site imaging data even when the biological covariate of interest (in this case age) is not balanced across sites [61, 62]. These site-corrected morphometric measures were used for estimation of morphometric similarity networks.

### Estimating Morphometric Similarity

Previously morphometric similarity was estimated from morphometric features measured in-vivo by both structural and diffusion MRI [28]. However, we highlighted significant correspondence between this morphometric similarity and that estimated with only features obtainable from a T1w MRI [27] and recent papers have similarly adopted this T1w-only approach [64], as does the current study.

To estimate morphometric similarity, the nodes for network construction were the ROIs from the Desikan-Killany atlas [33]. At an individual-level, the seven morphometric features estimated for each node can be expressed as a set of n vectors of length 10, with each vector as a different anatomical region (n = 68), and each element of the vector a different morphometric measure. To normalize measures within this length 10 vector, each morphometric feature is demeaned and SD scaled across the 68 regions, using Z-scores. A correlation matrix was generated for each participant, where each element of the matrix is the correlation between the feature vectors for every possible pairwise combination of regions. This correlation matrix represents the morphometric similarity derived meso-scale cortical organisation for each participant. This was an unthresholded matrix.

For each node/ROI, we calculated both nodal degree and nodal strength. Nodal degree was the number of edges that had survived thresholding for each node. Normalised nodal strength was calculated as the ‘magnitude’ of morphometric similarity for each node. This is defined as the sum of the MS weights of all of the edges of node *i*[65], normalised by the degree of the node (nodes with a higher number of edges will by definition have a greater magnitude of morphometric similarity). We also calculated the average nodal strength across the network to provide a global measure of the magnitude of morphometric similarity.

In subsequent exploratory analyses we investigated the thresholded matrix across multiple network densities (x = 5 to 40 in increments of 5), retaining only x% strongest absolute values of morphometric similarity across the graph. This has the effect of removing potential false-positive estimates of morphometric similarity. Metrics were calculated as per the unthresholded matrix.

### Sampling for Training, Validation and Testing Samples

The ABIDE cohort were divided into training, validation and test samples in the ratio of 5:1:2 (n = 204, n = 41 & n = 82 respectively). Sampling for the training sample was selected pseudo-randomly, via stratified under sampling based upon age. The entire sample was binned into 0.5 year bins dependent on age at scanning, up to the cutoff criteria of 17years. Bins for ages 6-9yrs were collapsed due to the much lower participant numbers in the lower tail of the age distribution. From each bin, 12 participants were randomly selected to derive the final training sample size. The remaining cohort was randomly split between validation and testing samples.

### Brain Age Prediction Models

Brain-age prediction was conducted across two, kernel-based regression approaches using the Kernlab package in R [66]; a) Gaussian Processes Regression (GPR) and b) Relevance Vector Regression (RVR). These were selected as these are both commonly used in the brain-age literature [9, 10]. Two different kernels were tested for each algorithm: a) laplacedot (Laplace radial basis kernel) and b) RBFDot (Gaussian radial basis function). Algorithm and kernel selection was conducted based upon performance on the validation set.

A GPR model was defined, with chronological age as the dependent variable and the morphometric data (for each of the feature sets) as the independent variables, to build a model of ‘healthy’ structural brain development. Prediction utilized 10 different feature-sets; i – vii) each of the individual morphometric features, viii) all features, ix) nodal-level strength of the MSN and x) edge-level weights of the MSN. Final model evaluation was conducted based upon performance on the test set.

In all cases, performance was evaluated based upon reducing mean absolute error (MAE) and maximizing predictive R^2^. Standard linear regression R^2^ is a biased estimate of model performance especially at at lower performances [67], whereas predicted R^2^ is more appropriate for quantifying regression accuracy [68], calculated as;

1 $$\mathrm{Pr}edictedR^{2}=1-NormalisedMSE$$

where the normalised MSE (Mean Squared Error) is equal to;

2 $$\mathrm{Pr}edictedR^{2}=1-\frac{MSE(\mathrm{Pr}edictedValue,observedValue)}{MSE(ObservedValue,MeanValue)}$$

## Robustness Of Brainage Models

### Robustness to Sampling

To assess robustness of models to the sampling partitions of the data, mean and confidence intervals of predictive R^2^ values are calculated. We carried out 100 random partitions (training/validation/testing) of the data and repeated analyses to generate a vector of 100 predictive R^2^ values for the testing set from which we can take a mean metric and assess the 95% confidence interval (see Table 4.).

### NHST of Models

To assess the ‘real effect’ of models in comparison to ‘null’ models, we used permutation testing to conduct null hypothesis significance testing (NHST). We established the null hypothesis as no meaningful patterns in the data between age and feature sets. To derive such models, we permuted (n = 1000) the dependent variable of age in the training and validation groups and reran the models. These were then tested on testing data where the true actual age was used. The mean predictive R^2^ values of the resampled models, and the distribution of R^2^ values from the permuted ‘null’ cases allowed calculation of *p*-values where the frequency of instances in the distribution where the mean predictive R^2^ was greater than that of the null models. Significance of p-values was assessed at the level of Bonferroni corrected α < 0.005, corrected over the 10 models.

## Figures and Tables

**Table 1:** Demographic information from entire cohort (n = 327 from ABIDE dataset) and the training/validation/test sets

	Entire Cohort	Training cohort	Validation cohort	Test cohort
n	327	204	41	82
Mean Age (yrs ± SD)	12.4 ± 2.5	12.7 ± 2.5	12.3 ± 2.6	11.8 ± 2.4
Min. Age (yrs)	6.5	6.5	7.3	7.3
Max. Age (yrs)	16.9	16.9	16.9	16.6
Sex (M:F)	259:68	164:40	31:10	64:18
Mean IQ (IQ ± SD)	110 ± 15^a^	110 ± 13^b^	111 ± 11^c^	109 ± 14^d^

a Available for n = 308,

b Available for n = 192,

c Available for n = 40,

d Available for n = 76

**Table 2:** Features sets used to produce Brainage models

Feature Set	N features
Individual Morphometric Features	
1. Surface Area	68
2. Curvature Index	68
3. Folding Index	68
4. Gaussian Curvature	68
5. Mean Curvature	68
6. Cortical Thickness	68
7. Cortical Volume	68
8. All Individual Features	476
Morphometric Similarity (MSN)	
9. MSN Nodal Strength	68
10. MSN Edge Weight	2278

**Table 3:** Performance of Brainage models trained on different feature sets and assessed on Training and Validation cohorts.

Feature	Algorithm	Kernel	Training		Validation		Feature	Algorithm	Kernel	Training		Validation	
			MAE (yrs)	Pred. R^2^	MAE (yrs)	Pred. R^2^				MAE (yrs)	Pred. R^2^	MAE (yrs)	Pred. R^2^
**Surface Area**	**GuassPrc**	**laplacedot**	**1.40**	**0.55**	**1.96**	**0.06**	**Cortical Thickness**	**GuassPrc**	**laplacedot**	**1.07**	**0.72**	**1.44**	**0.53**
		rbfdot	1.40	0.55	1.96	0.06			rbfdot	1.07	0.72	1.44	0.53
	RVM	laplacedot	1.51	0.45	2.28	−0.19		RVM	laplacedot	1.24	0.64	1.96	0.09
		rbfdot	1.47	0.48	2.23	−0.17			rbfdot	1.20	0.65	1.95	0.10
**Curvature Index**	**GuassPrc**	**laplacedot**	**1.40**	**0.57**	**2.06**	**0.02**	**Cortical Volume**	**GuassPrc**	**laplacedot**	**1.23**	**0.64**	**1.56**	**0.37**
		rbfdot	1.41	0.56	2.06	0.02			rbfdot	1.24	0.63	1.56	0.37
	RVM	laplacedot	1.41	0.49	2.54	−1.20		RVM	laplacedot	1.42	0.52	1.68	0.27
		rbfdot	1.28	0.57	2.59	−1.22			rbfdot	1.44	0.50	1.66	0.28
**Folding Index**	**GuassPrc**	**laplacedot**	**1.32**	**0.62**	**1.97**	**0.09**	**All Individual Features**	**GuassPrc**	laplacedot	1.05	0.74	1.37	0.49
		rbfdot	1.29	0.63	1.97	0.09			**rbfdot**	**1.06**	**0.73**	**1.37**	**0.49**
	RVM	laplacedot	1.32	0.52	3.22	−2.05		RVM	laplacedot	1.26	0.60	1.43	0.51
		rbfdot	1.39	0.48	3.02	−1.72			rbfdot	1.24	0.62	1.40	0.53
**Gaussian Curvature**	**GuassPrc**	**laplacedot**	**1.36**	**0.60**	**1.82**	**0.23**	**MSN: Nodal Strength**	**GuassPrc**	laplacedot	1.40	0.56	1.77	0.31
		rbfdot	1.34	0.61	1.82	0.23			**rbfdot**	**1.39**	**0.56**	**1.77**	**0.31**
	RVM	laplacedot	1.23	0.59	3.85	−3.41		RVM	laplacedot	1.32	0.57	2.01	0.05
		rbfdot	1.25	0.58	3.53	−3.01			rbfdot	1.18	0.64	2.03	0.03
**Mean Curvature**	**GuassPrc**	**laplacedot**	**1.40**	**0.56**	**1.91**	**0.19**	**MSN: Edge Weights**	**GuassPrc**	laplacedot	1.20	0.69	1.73	0.32
		rbfdot	1.42	0.55	1.91	0.19			**rbfdot**	**1.20**	**0.68**	**1.73**	**0.32**
	RVM	laplacedot	1.44	0.49	2.20	−0.10		RVM	laplacedot	0.36	0.96	1.82	0.31
		rbfdot	1.62	0.37	2.14	−0.04			rbfdot	0.33	0.97	1.82	0.31

Note. Pred. R^2^ = Predicted R^2^, GuassPrc = Guassian processes regression, RVM = Relevance Vector Machine, laplacedot = Laplace radial basis kernel, rbfdot = Gaussian radial basis function. Negative Pred. R^2^ values (in red) represent where performance was poorer than prediction using only the mean. **Bold** indicates for each feature set the combination of algorithm and kernel which produced the most favorable results in the validation set (based on predicted R^2^ as the evaluation metric).

**Table 4.** Performance of Brainage models trained on different feature sets and assessed on Training and Test samples.

Feature	Training		Test				
	MAE (yrs)	Pred. R^2^	MAE (yrs)	Pred. R^2^	Mean^a^ Pred. R^2^	Pred. R^2^ (95% CI^b^)	*p*-value^c^
Surface Area	1.41	0.54	1.93	0.04	0.04	(−0.06–0.15)	0.029
Curvature Index	1.39	0.57	1.91	0.02	−0.03	(−0.16–0.08)	0.233
Folding Index	1.35	0.60	1.92	0.06	0.04	(−0.11–0.15)	0.025
Gaussian Curvature	1.36	0.59	2.03	−0.05	0.02	(−0.09–0.13)	0.067
Mean Curvature	1.38	0.57	1.99	−0.09	0.01	(−0.10–0.12)	0.076
Cortical Thickness	1.07	0.72	1.48	0.37	0.41	(0.28–0.52)	**<0.001**
Cortical Volume	1.21	0.65	1.62	0.29	0.26	(0.13–0.38)	**<0.001**
All Individual Features	1.03	0.74	1.48	0.39	0.38	(0.27–0.47)	**<0.001**
MSN: Nodal Strength	1.35	0.58	1.94	0.02	0.10	(−0.02–0.23)	0.005
MSN: Edge Weights	1.16	0.70	1.73	0.19	0.23	(0.12–0.31)	**<0.001**

Note. Pred. R^2^ = Predicted R^2^, CI = Confidence Interval. Training sample represents the combination of both training and validation samples. Negative predicted R^2^ values (in red) represent where performance was poorer than prediction using only the mean.

a Mean and

b Confidence intervals of predictive R^2^ values are based upon 100 random partitions (training/validation/testing) of the data.

c *p*-value derived from 1000 permutations of age at scanning in the full training sample (see methods below, bold = significant at α < 0.05/10).

**Table 5:** Statistical associations between brainage Δ and covariates for each feature set

Covariate	n	Feature Set	Pearson		Spearman Rho	
			r	*P*	r	*P*
Age	82	Surface Area	**−0.94**	**<.00001**	**−0.94**	**<.00001**
		Curvature Index	**−0.96**	**<.00001**	**−0.95**	**<.00001**
		Folding Index	**−0.93**	**<.00001**	**−0.93**	**<.00001**
		Gaussian Curvature	**−0.95**	**<.00001**	**−0.94**	**<.00001**
		Mean Curvature	**−0.92**	**<.00001**	**−0.92**	**<.00001**
		Cortical Thickness	**−0.78**	**<.00001**	**−0.78**	**<.00001**
		Cortical Volume	**−0.90**	**<.00001**	**−0.89**	**<.00001**
		All Individual Features	**−0.90**	**<.00001**	**−0.89**	**<.00001**
		MSN: Nodal Strength^b^	**−0.95**	**<.00001**	**−0.93**	**<.00001**
		MSN: Edge Weights^b^	**−0.94**	**<.00001**	**−0.93**	**<.00001**
Motion^a^	82	Surface Area	−0.05	0.65	−0.16	0.16
		Curvature Index	−0.04	0.74	0.01	0.93
		Folding Index	0.06	0.57	0.04	0.71
		Gaussian Curvature	0.03	0.76	0.05	0.67
		Mean Curvature	0.01	0.94	0.04	0.75
		Cortical Thickness	−0.06	0.62	−0.08	0.48
		Cortical Volume	−0.23	0.04	−0.06	0.61
		All Individual Features	−0.07	0.51	−0.11	0.31
		MSN: Nodal Strength^b^	0.04	0.73	−0.08	0.47
		MSN: Edge Weights^b^	−0.03	0.77	−0.06	0.61
IQ^a^	56	Surface Area	0.23	0.08	0.24	0.08
		Curvature Index	0.10	0.47	0.09	0.50
		Folding Index	−0.05	0.72	−0.06	0.66
		Gaussian Curvature	0.03	0.83	−0.07	0.60
		Mean Curvature	−0.04	0.76	0.02	0.89
		Cortical Thickness	−0.04	0.77	0.00	0.99
		Cortical Volume	0.00	0.99	0.08	0.57
		All Individual Features	−0.14	0.30	−0.17	0.23
		MSN: Nodal Strength^b^	0.05	0.69	0.07	0.62
		MSN: Edge Weights^b^	−0.06	0.65	0.05	0.71
Covariate	n	Feature Set		Statistic		*P*
Sex^a^	82	Surface Area		−1.46		0.15
		Curvature Index		1.53		0.13
		Folding Index		0.33		0.74
		Gaussian Curvature		0.14		0.89
		Mean Curvature		1.25		0.22
		Cortical Thickness		−0.56		0.57
		Cortical Volume		1.64		0.10
		All Individual Features		−0.16		0.88
		MSN: Nodal Strength^b^		−0.39		0.70
		MSN: Edge Weights^b^		−0.32		0.75

Note.

a Controlling for actual age,

b derived from unthresholded MSN, **Bold** indicates those tests significant at bonferonni corrected α-level = .000833

## Data Availability

The data used in this research was acquired through the public Autism Brain Imaging Data Exchange (ABIDE, Di Martino, Yan [30]) database. Specifically, we used the ABIDE data release as shared by the Preprocessed Connectome Project (PCP, Bellec, Yan [31]. For full details see Pre-processed Connectome Project website http://preprocessed-connectomes-project.org/). The R code is also available from the authors upon request, however all open-source packages used in the study are listed here: *data.table, scales, psych, ggplot2, neuroCombat, ggseg, dplyr, ggpubr, ggExtra, kernlab, ppcor, PupillometryR, tidyr*.
